# Vitamin C

**DOI:** 10.1016/j.advnut.2023.100155

**Published:** 2023-11-21

**Authors:** Jens Lykkesfeldt, Anitra C Carr

**Affiliations:** 1 Faculty of Health and Medical Sciences, University of Copenhagen, Denmark; 2 Department of Pathology and Biomedical Science, University of Otago, Christchurch, New Zealand

Vitamin C is a small water-soluble carbohydrate; however, its particular chemical properties have rendered it as a unique reducing agent in living organisms. In all but a few species, ascorbic acid—the reduced form of vitamin C—is biosynthesized from glucose through a series of enzyme-catalyzed reactions. However, in higher-order primates, bats, guinea pigs, and some fish and bird species, this ability has been lost through evolution owing to accumulation of functional mutations and deletions in the gene encoding for l-gulonolactone oxidase, the enzyme catalyzing the final step in the biosynthesis of ascorbate. Consequently, vitamin C is an essential nutrient in these species and must be supplied through the diet to sustain life.

Vitamin C exists in 2 forms, the reduced form ascorbic acid and its two-electron oxidation product dehydroascorbic acid. All known biological functions of vitamin C are related to its reduced form. Thus, ascorbate can donate an electron to another molecule thereby reducing it, whereas itself being oxidized to the ascorbyl radical, a comparatively stable and nonharmful radical form. Two ascorbyl radicals can subsequently dismutate into 1 molecule of ascorbate and 1 molecule of dehydroascorbic acid. Dehydroascorbic acid is taken up by most cell types and efficiently reduced to ascorbate either chemically by glutathione or enzymatically by glutathione-dependent dehydroascorbic acid reductases or NADPH-dependent dehydroascorbic acid reductases such as thioredoxin reductase, thereby preventing metabolic loss of vitamin C. This process is referred to as ascorbate recycling.

Similar to its chemistry, the pharmacokinetics of vitamin C—that is, its absorption, distribution, metabolism, and excretion—is highly complex and tightly regulated by a range of mechanisms [1]. Ascorbate is taken up from the intestine through the sodium-dependent vitamin C transporter (SVCT) 1, an active transport mechanism capable of generating a concentration gradient. Dehydroascorbic acid may also be taken up from the gut to a minor extent by facilitated diffusion through glucose transporters. Distribution from the blood into tissues is governed by tissue-specific SVCT2s that concentrate vitamin C further from the typical 50–70 μmol/L in plasma to 0.5–10 mmol/L in tissues. The highest tissue concentrations of vitamin C are found in the brain, eyes, and adrenal glands. Vitamin C is excreted through the kidneys by glomerular filtration. However, if the body stores and plasma concentration are low, SVCT1 in the kidneys will almost quantitatively reabsorb ascorbate from the urine to prevent loss of vitamin C from the body. By contrast, if the vitamin C intake exceeds about 400 mg/d in healthy individuals over longer periods, the body becomes saturated, resulting in plateau plasma steady state concentrations of about 65–80 μmol/L, and any further excess of vitamin C is quantitatively excreted. Thus, this dose-dependent mechanism contributes to the homeostatic control of the vitamin C status of the body.

The biological functions of vitamin C are many and can roughly be separated into enzymatic and nonenzymatic reactions. The nonenzymatic reactions of ascorbate are what earned it its reputation as a powerful antioxidant. Ascorbate is indeed capable of reducing any pathophysiologically relevant free radical and reactive oxygen species. Vitamin C is also capable of regenerating the lipid-soluble vitamin E from its oxidized form. Through these activities and because of its abundancy, vitamin C is considered to be a very important contributor in the protection of cellular macromolecules such as DNA, proteins, and lipids from oxidative damage, although the clinical importance of this generic antioxidant activity is difficult to assess in vivo. Regardless, oxidative damage is believed to be causally related to both initiation and progression of several chronic diseases such as cardiovascular disease and cancer.

Along with its nonspecific antioxidant activity, more and more specific roles of vitamin C are being discovered. The longest known function of vitamin C is its role as cofactor for the ferrous and 2-oxoglutarate dependent dioxygenases that catalyze the hydroxylation of lysine and proline residues in unfolded procollagen chains to form the building blocks of the mature functional triple-helix collagen. Ascorbate is also a cofactor for enzymes involved in the biosyntheses of norepinephrine and carnitine, the amidation of peptide hormones, the metabolism of the amino acid tyrosine, the reduction of tetrahydrobiopterin, and the denitrosylation and phosphorylation of endothelial nitric oxide synthase. Through these actions, vitamin C facilitates a wide range of physiologic processes such as immune response, neurotransmission, energy metabolism, and vasorelaxation just to mention a few.

Ascorbate is also involved in the hydroxylation of hypoxia-inducible factor (HIF) 1α, which impacts the regulation of hundreds of genes and controls essential processes such as angiogenesis and cell proliferation. More recently, it has been discovered that ascorbate is also a cofactor for the Jumonji-C domain–containing histone demethylases and the ten-eleven translocation methylcytosine dioxygenases that are known as epigenetic master regulators. These enzymes catalyze the hydroxylation of methylated lysine and arginine residues in histones and methylated cytosine residues in DNA, which constitute the initial steps in demethylation that control gene expression. Through this activity, vitamin C seems to play an important role in normal epigenetic regulation and may also be important in disease prevention or treatment as acquired epigenetic changes are a hallmark of many cancers [2].

## Deficiencies

The ultimate clinical manifestation of vitamin C deficiency, scurvy, has been known for centuries from long sea voyages and expeditions and is fatal if not treated. The symptoms include impaired wound healing, gingivitis, perifollicular hemorrhages, ecchymoses, and petechiae and are largely related to impaired collagen formation and possibly HIF-1α hydroxylation. Clinical scurvy may be prevented with as little as 10 mg vitamin C per day. Other less-specific symptoms of severe and prolonged vitamin C deficiency include malaise and fatigue or lethargy and low mood. They may result from impaired energy metabolism and decreased neurotransmitter synthesis. Judging from the many specific functions of vitamin C, long-term insufficiency might also increase chronic disease risk. Indeed, a considerable body of epidemiologic literature has found significant inverse correlations between vitamin C status and risk of cardiovascular diseases and cancer. However, properly designed randomized controlled trials have so far not been conducted to confirm or reject a causal link between poor vitamin C status and increased risk of these diseases [3].

Poor vitamin C status is closely linked to a diet low in fresh fruits and vegetables and rich in fat and carbohydrates. Additional risk factors of vitamin C deficiency include smoking, pregnancy, low socioeconomic status, genetic predisposition, and several conditions related to cardiometabolic dysregulation such as hypertension, diabetes, and obesity [4].

## Dietary recommendation

Based on the vitamin C intake required to achieve near saturation of plasma and leukocytes with minimal urinary excretion and adjusted for body mass, a recommended dietary allowance (RDA) of 75 and 90 mg/d for females and males, respectively, was established in the most recent recommendation by the U.S. Institute of Medicine (now National Academy of Medicine) in 2000. In addition, the RDA for pregnant and breastfeeding females (19 y or older) was set at 85 and 120 mg/d, respectively. No RDA was established for infants; instead, the adequate intake of vitamin C was set at 40 mg/d for infants aged younger than 6 mo and 50 mg/d for infants aged 6–12 mo. For older children, the recommendation was based on estimated body mass in relation to an adult: 15 mg/d for children younger than 3 y, 25 mg/d for children younger than 8 y, and 45 mg/d for children younger than 13 y. Moreover, the RDA for teenagers was based on gender: 75 and 65 mg/d for boys and girls aged 13–17 y, respectively. Smokers are known to have an increased turnover of vitamin C probably owing to the toxicity of the smoke and consequently an additional intake of 35 mg/d is recommended [5]. Although environmental tobacco smoke exposure has a similar effect, no recommendations have been put forward for those exposed to smoking.

In a more global perspective, recent reports shows that considerable discrepancies exist between the recommendations published by national authorities. The differences are typically based on the health perspective underlying the recommendations. Thus, scurvy prevention can be achieved with a very small intake of vitamin C and has been the basis for the recommendations by, for example, WHO of only 45 mg/d, whereas some countries (Australia, New Zealand, and China) have introduced a suggested dietary target of 200 mg/d with the aim of optimal chronic disease risk reduction [6]. Moreover, with the ongoing obesity pandemic, evidence suggests current recommendations are too low for a large proportion of the population and that body weight should be considered in future dietary recommendations because vitamin C status is strongly inversely correlated with body weight [4].

## Food sources

Most fruits and vegetables are excellent sources of vitamin C, although considerable variation exists. Citrus, kiwi, and mango fruits and vegetables such as peppers and broccoli are rich in vitamin C, while apples, bananas, and most staple foods have low vitamin C content. Vitamin C content may be severely decreased during heating and storage. Five to 9 servings of fresh, frozen, or minimally processed fruits and vegetables has been estimated to correspond to about 200 mg of vitamin C.

## Clinical uses

Although scurvy is easily prevented even by a low intake of vitamin C, symptoms of scurvy typically reflect a profound deficiency that requires urgent parenteral replacement therapy. Subsequently or in milder cases, oral supplementation with 500 mg/d may be adequate. Subclinical vitamin C deficiency is very difficult to detect without blood sampling owing to the often-unspecific symptoms. Although overt vitamin C deficiency is rare in the general population, increased frequencies may be found among individuals with malnutrition, poor dietary habits, chronic disease, intestinal disorders, or chemical dependencies. Clinical uses of vitamin C also include increasing nonheme iron absorption because vitamin C reduces dietary iron and facilitates its intestinal absorption.

Vitamin C is currently under investigation as a therapy in several clinical conditions such as cancers, sepsis, cardiovascular disease, and coronavirus infections. However, no final conclusion has been reached on the relevance of clinical application of vitamin C beyond that of replacement therapy during acute disease. Of note, however, the doses necessary to restore normal levels in acutely ill patients are usually many-fold higher than those required in healthy individuals.

## Toxicity

Vitamin C is generally nontoxic and well tolerated even in large doses. The current tolerable upper intake level was set to 2 g/d by the National Academy of Medicine in 2000, but more recently, the European Food and Safety Administration (EFSA) and others have removed the upper intake level for vitamin C completely owing to a lack of evidence of toxicity. Temporary gastrointestinal disturbances are known to occur in some individuals at higher doses, and a consistent intake of several grams per day has been suspected of increasing risk of kidney stones, but this suspicion has not been substantiated. However, those susceptible to kidney stone formation are still recommended to avoid vitamin C supplements. Patients at risk of iron overload should limit iron intake rather than that of vitamin C.

## Recent research

Although vitamin C has been investigated as a potential cancer therapy for decades, the recent identification of its involvement in epigenetic regulation as mentioned earlier has revealed a new and highly plausible mechanism by which vitamin C deficiency may contribute to cancer progression and supports a possible therapeutic potential. Studies investigating the effect of vitamin C supplementation on progression-free survival are currently underway [2]. Vitamin C’s immune-modulating effect has also been of interest for decades for its possible ability to attenuate the common cold. More recently, more clinically important applications in relation to sepsis and serious SARS-CoV-2 infections have been investigated but so far with mixed outcomes. However, numerous trials are still ongoing and may provide more definitive results.

### Author contributions

JL: wrote the manuscript; ACC: critically edited the manuscript. Both authors read and approved the final manuscript.

### Conflict of interest

The authors report no conflicts of interest.
